# Prevalence of Smoke-Free Car and Home Rules in Maine Before and After Passage of a Smoke-Free Vehicle Law, 2007–2010

**DOI:** 10.5888/pcd11.130132

**Published:** 2014-01-16

**Authors:** Rebecca Murphy-Hoefer, Patrick Madden, Dorean Maines, Carol Coles

**Affiliations:** Author affiliations: Patrick Madden, Market Decisions, Portland, Maine; Dorean Maines, Carol Coles, Partnership for a Tobacco-Free Maine, Augusta, Maine.

## Abstract

**Introduction:**

This is the first study to examine the prevalence of self-reported smoke-free rules for private cars and homes before and after the passage of a smoke-free vehicle law.

**Methods:**

Data were examined for 13,461 Maine adults aged 18 or older who participated in the Behavioral Risk Factor Surveillance System, a state-based telephone survey covering health topics. Self-reported smoke-free car and home rules, smoking behavior, and demographic variables of age, sex, education, income, and children in household were analyzed for prevalence before and after the state’s smoke-free vehicle law was passed.

**Results:**

Prevalence of smoke-free car and home rules was significantly higher after Maine’s smoke-free vehicle law was passed in the state (*P* = .004 for car rules and *P* = .009 for home rules). Variations in smoking rules differed by smoking and demographic variables. People with household incomes of less than $20,000 saw an increase of 14.3% in smoke-free car rules; overall, those with annual incomes of less than $20,000 and those with less than a high school education reported a lower prevalence of smoke-free car rules both before and after the law was passed than did people with higher incomes and higher education levels. The prevalence of smoke-free home rules after the law was implemented was higher among those with 4 or more years of college education than among those with lower levels of education (*P* = .02).

**Conclusion:**

The prevalence of smoke-free car and home rules among Maine adults was significantly higher after the passage of a statewide smoke-free vehicle law. This apparent change in smoke-free rule prevalence may be indicative of changing social norms related to the unacceptability of secondhand smoke exposure.

## Introduction

In the United States, 88 million nonsmokers were exposed to secondhand smoke in 2007 through 2008, including 32 million children aged 3 to 19 years (1). The involuntary exposure to tobacco smoke among infants and children increases risk of illness and premature death (2,3). Given that small children breathe at a faster rate than adults, they are exposed to a greater quantity of air pollutants, including cigarette smoke (4). Smoking inside a small enclosed space such as a car is particularly dangerous because the concentration of pollutants from cigarette smoke can accumulate rapidly (5). Vehicle-related exposure to tobacco smoke causes infants to be lethargic (6) and young people to be twice as likely to develop asthma (7). Even with ventilation in vehicles, secondhand smoke from 1 or more cigarettes exceeds Environmental Protection Agency (EPA) limits (6,8). The 2006 Surgeon General’s report concluded that there is no safe level of exposure to secondhand tobacco smoke (2).

Although comprehensive state laws that ban smoking in public and private worksites, restaurants, and bars have increased, covering nearly half of US residents (9,10), these laws do not protect children from exposure in private areas, such as vehicles and homes (4). In Maine, 22.8% of adults aged 18 or older currently smoke (9). As a result, 53% of Maine high school students and 41% of middle school students are exposed to secondhand smoke at least once during any given week (11). The city of Bangor was the first and only locality in Maine to pass a smoke-free vehicle ordinance, which went into effect in 2007 (12). In January 2008, Maine passed a statewide law prohibiting smoking in a motor vehicle by the operator or a passenger whenever a person under the age of 16 years is present (13). This law went into effect on September 1, 2008.

Six states and 1 territory have passed laws prohibiting smoking in vehicles occupied by children (14). This is the first study to examine the prevalence of smoke-free car rules and smoke-free home rules before and after the passage of a smoke-free vehicle law. The objective of our study was to examine whether there was a difference in self-reported, smoke-free car and home rules after the passage of Maine’s smoke-free vehicle law. The secondary purpose was to examine the characteristics of those who reported having such rules.

## Methods

The Behavioral Risk Factor Surveillance System (BRFSS) is a cross-sectional state-based telephone health survey conducted through a random-digit–dialing methodology among persons aged 18 years or older (15). To study the prevalence of current cigarette smoking and the proportion of those with smoke-free car rules in Maine, BRFSS data from 2007 through 2010 were examined. Beginning in 2007, the Maine BRFSS partnered with the Maine Department of Health and Human Services, Maine Center for Disease Control and Prevention’s Partnership for a Tobacco Free Maine program for a BRFSS split survey. In this split survey, the Centers for Disease Control and Prevention’s core questions (called Part A) are the same in each survey, and the state adds questions (called Part B) that differ from state to state.

The sampling frame for Maine’s Part B questions included only landline respondents from 2007 to 2010. The year 2007 is the first year data were collected for Maine’s Part B questions, and no data on smoke-free rules from BRFSS exist before that time. A cellular telephone sample and changes to the weighting methods were introduced in 2011 and, because of these changes, data after 2010 are excluded from this analysis. The overall sample size for the Part B survey ranged from 2,648 in 2007 to 4,072 in 2010; Part B had a total sample size of 13,461 over the 4 years. Response rates ranged from a low of 47.8% in 2007 to a high of 58.3% in 2010.

To examine the impact of Maine’s smoke-free vehicle law from 2007 through 2010, we separated BRFSS Part B surveys into 2 groups for analysis: 1) surveys collected in 2007 and 2) those collected after passage of the law from 2008 through 2010. The final sample sizes in this analysis were 2,648 respondents for the 2007 segment and 10,813 for the 2008 through 2010 segment.

Data from the BRFSS Part B surveys were analyzed, including demographic variables of age, sex, education, annual household income, number of children in the household, and smoking status. Differences between reported smoking rules before and after the law was passed were tested for significance overall and within each demographic category.

Participants were asked the following questions from the BRFSS core questions to determine cigarette smoking prevalence: “Have you smoked at least 100 cigarettes in your entire life?” and “Do you now smoke cigarettes every day, some days, or not at all?” Current smokers were defined as those who reported having smoked 100 or more cigarettes during their lifetime and who currently smoke every day or some days.

Data from the following BRFSS secondhand smoke survey items in Part B were examined: “Which of the following statements best describes the rules about smoking inside your car? No one is allowed to smoke inside your car, smoking is not allowed if children are in your car, or smoking is permitted anytime inside your car.” and “Which of the following statements best describes the rules about smoking inside your home? No one is allowed to smoke anywhere inside your home, smoking is allowed in some places or at some times, smoking is permitted anywhere inside your home.” All analyses and statistical tests were conducted using SUDAAN (Research Triangle Institute, Research Triangle Park, North Carolina), a statistical software package for the analysis of correlated data, including correlated data encountered in complex sample surveys like the BRFSS. Two-tailed *t* tests for differences in proportions were conducted in SUDAAN to examine whether the policies of smoking inside cars or homes 1) changed after the passage of Maine’s smoke-free vehicle law and 2) are significantly different within each of the demographic characteristics listed above.

## Results

Among all participants in the BRFSS Part B survey from 2007 through 2010 (n = 13,461), slightly less than half were male (48.2%) (Table 1). The mean age of participants was 48.6 years, and the range of ages was from 18 to 97 years. The median annual household income among survey participants was between $35,000 and $49,999. Approximately one-third of the survey population (32.1%) completed high school or had a general equivalency diploma (GED), and 6.6% had less than a high school education. One-quarter (25.7%) attended some college or had a technical degree, and the remainder (35.6%) had a college degree or higher. Close to two-fifths of participants (37.6%) had at least 1 child in their household. Approximately half (49.3%) of the survey population had smoked at least 100 cigarettes in their lifetime. Overall, at the time of the surveys, more than four-fifths (82.4%) did not smoke, and 17.6% were current smokers. All demographics showed nonsignificant change between the 2007 survey and the 2008 through 2010 surveys at *P* < .05.

**Table 1:** Characteristics of Respondents to Maine’s Behavioral Risk Factor Surveillance System (BRFSS) Part B Before and After Passage of a Law Prohibiting Smoking in Vehicles, 2007–2010^a^

Characteristic	Before Law (2007)		After Law (2008–2010)^b^		Combined 2007–2010	
	Unweighted Sample (n = 2,648)^c^	Weighted %^d^	Unweighted Sample (n = 10,813)^c^	Weighted %^d^	Unweighted Sample (n = 13,461)^c^	Weighted %^d^
**Sex**						
Male	964	48.2	4,206	48.2	5,170	48.2
Female	1,684	51.8	6,607	51.8	8,291	51.8
**Age, y**						
18–24	76	11.9	295	9.1	371	9.8
25–34	243	13.9	692	14.3	935	14.2
35–44	417	18.2	1,453	19.4	1,870	19.1
45–54	590	20.7	2,321	20.8	2,911	20.8
55–64	606	16.2	2,620	16.9	3,226	16.8
≥65	700	19.0	3,355	19.5	4,055	19.4
**Education**						
Less than high school diploma	189	7.6	696	6.3	885	6.6
High school graduate or GED	804	30.5	3,512	32.6	4,316	32.1
Some college or technical school	684	27.3	2,659	25.1	3,343	25.7
College 4 years or more	968	34.6	3,933	36.0	4,901	35.6
**Annual household income, $**						
<20,000	454	16.3	2,002	16.6	2,456	16.5
20,000–34,999	524	22.2	2,129	20.1	2,653	20.6
35,000–49,999	412	17.8	1,650	17.5	2,062	17.6
50,000–74,999	408	18.3	1,618	18.8	2,026	18.7
≥75,000	519	25.3	2,205	27.0	2,724	26.6
**Children in household**						
Yes	709	35.6	2,743	38.3	3,452	37.6
No	1,936	64.4	8,061	61.7	9,997	62.4
**Current smoking status**						
Current smoker	475	19.0	1,684	17.1	2,159	17.6
Nonsmoker	2,160	81.0	9,064	82.9	11,224	82.4

Abbreviations: BRFSS, Behavioral Risk Factor Surveillance System; GED, general equivalency diploma.

a Part B is survey questions added by the state to the core BRFSS survey questions.

b No significant differences in demographics from before to after the law was enacted at *P* < .05.

c Missing values are not included.

d Percentage of valid responses among respondents in each category.

### Smoke-free car rules

Table 2 shows the relative change in smoke-free car rules by various demographic factors after the smoke-free vehicle law was passed. Overall, 78.8% of Maine adults aged 18 years or older reported a smoke-free car rule after the smoke-free vehicle law was passed. This is a significant change from before the law, when 74.9 of adults reported having a smoke-free car rule (*P* = .004). The percentage of adults reporting no rule (ie, smoking is allowed anytime inside car) was significantly lower after the law was in place (from 7.9% before the law to 5.1% after the law, *P* < .001). Examining the results by year shows no significant difference between reported rules in the 3 years after the law was implemented (78.6% in 2008, 78.1% in 2009, and 79.8% in 2010), an indication that the prevalence of smoke-free car rules was consistent in the 3 years following passage of the law.

**Table 2:** Adults Aged 18 or Older Reporting Smoke-Free Car Rule (No One Is Allowed to Smoke Inside the Car) Before and After Passage of a Law Prohibiting Smoking in Vehicles, Behavioral Risk Factor Surveillance System, Maine, 2007–2010

Characteristic	Before Law (2007)		After Law (2008–2010)		% Change	*P* Value^b^
	Unweighted Sample Size^a^	% With Rule	Unweighted Sample Size^a^	% With Rule		
**Overall**	2,399	74.9	9,960	78.8	5.2	.004
**Year**						
2007	2,399	74.9	NA		NA	
2008	NA		2,498	78.6		
2009			3,697	78.1		
2010			3,765	79.8		
**Sex**						
Male	867	72.6	3,876	75.6	4.1	.17
Female	1,532	77.0	6,084	81.8	6.2	.005
**Age, y**						
18–24	66	56.2	255	63.7	13.3	.33
25–34	218	74.6	653	76.6	2.7	.61
35–44	381	70.5	1,365	75.6	7.2	.09
45–54	541	75.1	2,140	77.8	3.6	.28
55–64	558	80.4	2,448	81.7	1.6	.52
≥65	622	85.5	3,033	88.9	4.0	.05
**Education**						
Less than high school diploma	149	56.9	563	64.5	13.4	.30
High school graduate or GED	721	65.6	3,162	71.1	8.4	.04
Some college or tech school	614	76.3	2,468	77.8	2.0	.56
College 4 years or more	913	85.0	3,754	88.2	3.8	.05
**Annual household income, $**						
<20,000	373	56.9	1,695	65.0	14.2	.05
20,000–34,999	487	70.9	1,984	73.8	4.1	.41
35,000–49,999	387	74.5	1,576	75.2	0.9	.83
50,000–74,999	385	77.0	1,547	81.6	6.0	.16
≥75,000	491	86.4	2,113	88.8	2.8	.27
**Children in household**						
Yes	639	69.2	2,577	77.2	11.6	.002
No	1,758	78.0	7,375	79.8	2.3	.24
**Current smoking status**						
Current smoker	398	21.8	1,454	26.9	23.4	.14
Nonsmoker	1,991	86.4	8,454	88.5	2.4	.09
**Smoking status and children in household**						
Current smoker and children in household	132	19.2	457	30.7	59.9	.05
Current smoker and no children in household	266	23.8	997	24.0	0.8	.84

Abbreviations: NA, not applicable; GED, general equivalency diploma.

a Missing values are not included.

b Two-tailed *t* tests for differences in proportions.

By demographic characteristics (Table 2), significant changes in reported smoke-free car rules occurred after the law was passed compared with before the law was passed among women (prevalence was 6.2% higher, *P* = .005), high school graduates and those with a GED (8.3% increase, *P* = .04), and those with children in their household (11.6% increase, *P* = .002). Although adults with annual household incomes of less than $20,000 had the highest percentage increase (14.2%) in smoke-free rules after the law was passed compared with before the law, both those with incomes of less than $20,000 and those with less than a high school education had a lower prevalence of smoke-free car rules before and after the law was passed than did those with incomes above $20,000 and those with a high school education or better. Only 64.5% of adults with less than a high school education reported having a smoke-free car rule after the law was implemented, but 88.2% of college graduates reported having such a rule. Among adults with household incomes of less than $20,000, 65.0% had a smoke-free car rule after the law was implemented, compared with 88.8% of those with annual household incomes of $75,000 or more.

Both before and after passage of the law, nonsmokers were more likely to report having a voluntary smoke-free car rule (86.4% before and 88.5% after) than were current smokers (21.8% and 26.9%) (*P* < .001). Although not significant, this 23.3% relative increase for current smokers is the largest for any demographic groups; however, current smoker sample sizes in the BRFSS are smaller than many other demographic groups, which may contribute to the lack of statistical power for the current smoker segment. Among adults who never smoked, 9 in 10 reported having a smoke-free car rule after the law was implemented.

Among respondents who had children in their household and who reported not allowing smoking in their car, the prevalence was significantly higher after the law was passed (69.2% before and 77.2% after, *P* = .002) (Table 2). In addition, the percentage of those who allowed smoking any time in the car was significantly lower (reduced by nearly half) after implementation of the law. Only 3.7% of adults with children in their household allowed smoking anytime in their cars after the law was implemented compared with 6.8% before the law was implemented (*P* = .04). Although not significant, there was an increase in the percentage of current smokers with children in the household who reported having a smoke-free car rule, from 19.2% before the law was passed to 30.7% after the law was passed; this 59.9% relative increase is the largest among all of the demographic groups. By contrast, adult smokers without children showed no significant change in the percentage who reported having rules about not smoking in their cars after the law went into effect (23.8% before; 24.0% after).

A secondary analysis comparing the period before the law’s implementation and the period afterward (ie, comparing January 2007 through August 2008 with September 2008 through December 2010) revealed that smokers with children in their household reported significantly higher levels of smoke-free car rules after the law was implemented (31.5%) than before implementation (22.0%) (*P* = .03), a percentage increase of approximately 43% (data not shown).

### Smoke-free home rules

The percentage of respondents reporting having a smoke-free home rule was significantly higher after passage of the smoke-free car law, 83.1%, than before, 79.9%, *P* = .009 (Table 3). The prevalence of smoke-free home rules after the law was implemented was higher among those with 4 or more years of college education than among those with lower levels of education (*P* = .02).

**Table 3:** Percentage of Adults Aged 18 or Older Reporting Smoke-Free Home Rule (Smoking Is Not Allowed Anywhere Inside the Home) Before and After Passage of a Law Prohibiting Smoking in Vehicles, Maine, 2007–2010

Characteristic	Before Law (2007)		After Law (2008–2010)		% Change	*P* Value^b^
	Unweighted Sample Size^a^	% With Rule	Unweighted Sample Size^a^	% With Rule		
**Overall**	2,488	79.9	10,277	83.1	4.0	.009
**Year**						
2007	2,488	79.9	NA		NA	
2008	NA		2,590	83.0		
2009			3,821	83.3		
2010			3,866	82.9		
**Sex**						
Male	899	79.0	3,973	82.0	3.8	.11
Female	1,589	80.7	6,304	84.1	4.2	.04
**Age, y**						
18–24	72	73.9	277	77.4	4.7	.62
25–34	225	85.9	660	88.2	2.7	.45
35–44	390	80.6	1,391	85.3	5.8	.07
45–54	552	80.1	2,202	81.7	2.0	.47
55–64	577	78.5	2,510	81.7	4.1	.14
≥65	658	78.9	3,169	82.5	4.6	.08
**Education**						
Less than high school graduate	175	60.7	635	65.4	7.7	.49
High school graduate or GED	748	75.4	3,300	76.2	1.1	.74
Some college or tech school	637	79.4	2,547	84.3	6.2	.05
College 4 years or more	925	88.1	3,782	91.2	3.5	.02
**Annual household income, $**						
<20,000	417	65.8	1,892	68.3	3.8	.50
20,000–34,999	498	74.7	2,019	78.9	5.6	.21
35,000–49,999	393	79.9	1,595	81.0	1.4	.70
50,000–74,999	390	82.5	1,550	90.0	9.1	.009
≥75,000	491	90.6	2,112	91.8	1.3	.48
**Children in household**						
Yes	648	85.7	2,619	88.5	3.3	.18
No	1,838	76.8	7,650	79.7	3.8	.05
**Current smoking status**						
Current smoker	433	50.5	1,571	54.9	8.7	.24
Nonsmoker	2,043	86.5	8,651	88.7	2.5	.08
**Smoking status and children in household**						
Current smoker and children in household	139	66.5	479	71.4	7.4	.44
Current smoker and no children in household	294	38.7	1,092	42.9	10.9	.36

Abbreviations: BRFSS, Behavioral Risk Factor Surveillance System; GED, general equivalency diploma.

a Missing values are not included.

b Two-tailed *t* tests for differences in proportions.

## Discussion

Our study shows that the prevalence of smoke-free car and home rules among Maine adults was significantly higher after the passage of its statewide smoke-free vehicle law. The fact that this change in smoke-free rule prevalence coincided with the passage of a smoke-free vehicle law may be indicative of changing social norms related to the unacceptability of secondhand smoke exposure among Maine adults. The link between public smoke-free laws and voluntary smoke-free rules is a possible explanation for the change that was observed in our study (16).

The intent to protect children is demonstrated by the increase in smoke-free car rules after the passage of the law. Current smokers with children in their household reported an increase in smoke-free rules after the law was passed compared with almost no increase for smokers without children in their household. However, disparities exist as seen in the literature (17,18). Another smoke-free vehicle study found misperceptions among low-income adults regarding the efficacy of keeping windows down to avoid the hazards of exposure to secondhand smoke (19).

Although smoke-free car rules increased in Maine, more work remains to be done. Among all participants surveyed from 2007 through 2010, 16.4% still allowed smoking when children were not present. This is problematic because the residues from tobacco smoke components stay on vehicle surfaces. One study found that used vehicles owned previously by smokers had significantly higher levels of nicotine in the air, dust, and vehicle surfaces than vehicles owned by nonsmokers (20). Another study detected the interaction of cigarette smoke and vehicle engine emissions forming carcinogenic tobacco-specific nitrosamines (21). This finding is cause for action given the harm to infants and children because of their contact with surfaces and quicker respiration (4).

Children are among the most exposed groups to secondhand smoke (1). Our study also reviewed smoke-free home policies during the same period, because children are more likely than nonsmoking adults to live with someone who smokes. Concern over a potential increase in smoking in homes after the implementation of smoke-free laws in public places has been discussed and found to be unwarranted (16). Our results also counter this concern given that voluntary smoke-free home policies also increased significantly after Maine’s law was implemented. Because smoke-free home rules are voluntary, they serve as an indicator of public attitudes and social norms, as well as support for smoke-free indoor environment policies (16,22–24). Other studies found that smoke-free home policies reduce smoking initiation among young people and help smokers quit (2). The implications for public health are immense; childhood exposure to secondhand smoke increases the risk of illness and premature death (2,3), including respiratory diseases (2); sudden infant death syndrome (SIDS) (2); cancer as an adult (25); higher lead blood levels (26); and attention-deficit/hyperactivity disorder, learning disabilities, and conduct disorders (27). Among US children, secondhand smoke exposure costs $4.98 billion in annual health care expenditures (28).

Possible explanations for the positive outcomes of Maine’s state law include the comprehensive programs conducted throughout the state. In particular, to educate the public about the law in Maine and to raise awareness about the effects of secondhand smoke exposure, the Partnership for a Tobacco-Free Maine implemented a statewide health communication campaign titled, “Wherever You Live and Breathe, Go Smoke-Free.” The campaign included the use of television and radio. The media campaign was on air from February 14, 2008, through November 30, 2008. Furthermore, Maine has strong smoke-free workplace and public place laws that contain few exceptions or loopholes. Maine does not prevent localities from implementing policies stronger than the state laws, and many localities have done so. Support for the current law may continue because research shows that more favorable attitudes toward smoke-free laws exist as a result of advances in the scope of smoke-free indoor environment policies (16,29), including policies related to smoking in vehicles (30).

A limitation of this study is that estimates for cigarette smoking and voluntary smoke-free rules are based on self-reports and are not validated with biochemical tests. However, self-reported data on current smoking status have high validity (15). Another limitation is that the Maine BRFSS survey item includes smoke-free rules for cars, not all types of vehicles such as sport-utility vehicles, minivans, and trucks. As a result, the current prevalence data may be a conservative number.

The smoke-free car law is part of a comprehensive tobacco control program that includes multiple interventions: state and community, health communication, and tobacco cessation. Therefore, it is difficult to separate the effects of 1 aspect of a comprehensive program from all other aspects. In particular, the statewide “Wherever You Live and Breathe, Go Smoke-Free” campaign may have contributed to the increase in voluntary smoke-free car and home rules. Other factors that may account for the increase in voluntary home rules could be the federal cigarette tax that went into effect in 2009 and the trend toward lower smoking prevalence in Maine.

Because of the limited availability of data, equivalent survey measures between 2007 and 2010 were not available to make comparisons with an analogous state that does not have a smoke-free car law; however, we found 2 data sources that may provide support for our findings. Vermont has similar statewide laws prohibiting smoking in public places and worksites, but not a smoke-free car law. In 2007 Maine and Vermont had a statistically equivalent prevalence of voluntary smoke-free home rules, 75.4% in Vermont and 76% in Maine according to the Tobacco Use Supplement to the Current Population Survey (TUS-CPS) (31). In 2010, Maine had a significantly higher prevalence of voluntary smoke-free home rules than Vermont (Maine 84.4% vs Vermont 80.6%), according to the National Adult Tobacco Use Survey (2010 TUS-CPS data were not available when we wrote this article) (9). It is a limitation that these data are from 2 different sources; however, these were the only data available to provide additional insight. These data sources as well as year intervals are the same as those featured in the Centers for Disease Control and Prevention’s Tobacco Control State Highlights 2010 (9,31). There are limited data about smoke-free vehicle rules and consistent survey questions. Consideration of smoke-free vehicle rule measures in surveillance surveys are needed to conduct future studies to compare demographics and smoke-free vehicle rules among states.

Although exposure to tobacco smoke has declined in the United States, much remains to be done to reduce the disparities in exposure, especially among young people. In addition to protecting children, smoke-free vehicle laws may also reduce the negative health consequences experienced by other vulnerable populations and all nonsmokers given that there is no safe level of exposure to secondhand smoke (2). States with comprehensive smoke-free indoor policies that include worksites such as restaurants and bars should consider extending them to include private areas such as vehicles. Smoke-free vehicle laws may strengthen support for the protection of populations from exposure to secondhand smoke, increase the population’s knowledge about the negative effects of secondhand smoke, increase smoke-free norms, and assist in reducing tobacco use.
